# The inhibiting effects of resistance to change of disability determination system: a status quo bias perspective

**DOI:** 10.1186/s12911-020-1090-7

**Published:** 2020-04-29

**Authors:** Wen-Chou Chi, Po-Jin Lin, I-Chiu Chang, Sing-Liang Chen

**Affiliations:** 1 Department of Occupation Therapy, Chung Shan Medical University, Occupational Therapy Room, Chung Shan Medical University Hospital, No.110, Sec.1, Jianguo N.Rd, Taichung City, 40201 Taiwan, Republic of China; 2 Department of Information Management, National Chung Cheng University, No.168, Sec. 1, University Rd., Minhsiung, Chiayi, 62102 Taiwan, Republic of China; 3 Department of Information Management, Nanhua University, No.55, Sec. 1, Nanhua Rd., Dalin Township, Chiayi County, 62249 Taiwan, Republic of China

**Keywords:** Status quo bias, Technology acceptance model, Resistance to change

## Abstract

**Background:**

Information systems implementation projects have been historically plagued by failures for which user resistance has consistently been identified as a salient reason. Most prior studies investigated either the causes or the consequences of Resistance to Change (RTC) of medical related Information Systems. In this study, we simultaneously explore the causes and impacts of RTC of Disability Determination System (DDS).

**Methods:**

This study adopts the Status Quo Bias perspective and combines the information systems usage model and Technology Acceptance Model (TAM) as theoretical foundation to investigates the causes and impacts of users’ RTC on their intention to use the DDS. Data were obtained through internet questionnaire survey. Totally, 326 respondents from 22 local governments and 142 hospitals were collected, of which 252 were valid samples and were analyzed using structure model analysis.

**Results:**

The research model is proved with eight out of 11 hypotheses being supported. The antecedents of RTC can explain 21.4% of the RTC variation, and the RTC impacts can explain 57.5% of the variation of intention to use DDS.

**Conclusions:**

Combining the Status Quo Bias perspective and key component of TAM provides an adequate explanation of adopting intention of changing systems and extend the existing knowledge of information systems adoption. The results provide as a reference for managing users’ RTC and enhance the effects and efficiency of new systems adoption.

## Background

Organizations have continued to increase their investment in information technology (IT) and information systems (IS). Despite the huge investment costs, many researchers indicate that the failure rates on IT/IS projects are unacceptably high, thus raising serious concerns regarding the successful implementation of the projects. User resistance has been consistently identified as the contributing factor of implementation failures [1]. In the medical context, the benefits of electronic health record systems are not always realized because of the various difficulties associated with them. Similarly, many of these failures are traced back to user resistance [2].

This study was motivated by the lack of information about the characteristics of Taiwan’s frontline medical social service personnel, a section of employees who deal primarily with the disabilities. When the disability determination system (DDS) was implemented, there were many complaints from users, and 10% of the users working for the local government changed their job positions just to avoid using the system. A considerable amount of time and money were spent because many users were against using the system. In order to clarify the users’ resistance of the DDS, the causes and impacts of resistance are needed to be concerned simultaneously.

The antecedents of resistance to change are explained by the status quo bias in three main categories: rational decision making, cognitive misperceptions, and psychological commitment. This research combines the IS usage model, Technology Acceptance Model (TAM), and the status quo bias perspective to examine the causes behind user resistance, to analyze the inhibitor effects, and to validate the relationships among the variables and user intention with empirical data of frontline medical/social service staff using the DDS. The results of this study can be used to manage the resistance for better acceptance of DDS and contribute to the knowledge of IS adoption.

### Resistance to change (RTC)

Resistance is used to describe a wide variety of phenomena at all levels of human social life and in a number of different settings, including political systems, entertainment, literature, and the workplace. The behavioral theory of user resistance is based on the perspective of RTC and is initiated from a series of studies on “organization change and change management” [3]. RTC has been studied as a construct in various fields. Studies of user RTC in the context of IS were more conceptual [4] and few studies included quantitatively validating, such as survey-based research or experimental data, on how users develop RTC related to new IS implementation [5, 6]. Some believe that previous studies have narrowly focused on finding the causes of user resistance [1].

### Status quo bias (SQB)

The SQB perspective assumes that individual decision makers are biased toward maintaining the status quo, that is, “doing nothing or maintaining one’s current or previous decision” and explains why individuals make a disproportionate number of decisions to continue with an old status rather than switching to a new action [7]. Meanwhile, switching costs including uncertainty costs, transition costs, and sunk costs, and can be measured by time, effort, and money value. The SQB perspective provides a base of the causes of RTC, therefore using SQB as antecedents to explain RTC is more theoretical than arbitrarily selecting any variables. They also categorize the SQB perspective into three main categories: rational decision making, cognitive misperceptions, and psychological commitment. Rational decision making refers to an assessment of the relative costs and benefits of change before making a decision related to a new action. Rational decision making alone does not completely explain SQB, as it may also be the result of cognitive misperceptions that generate from loss aversion [7]. This implies that people tend to weigh latent losses as being greater than latent gains in making decisions as to whether they should move away from the status quo [8]. The last explanatory category of SQB refers to psychological commitment. Sunk cost, social norms, and efforts to feel in control are the three main factors that contribute to psychological commitment [7]. This desire can lead to SQB because people do not want to lose control when switching to an unknown system or unknown way of working.

Many studies confirmed the SQB perspective and suggested reasons for user resistance were negative transitions entailing a cost, such as loss of power [9]; expending more effort because of poor system quality or changes in the nature of the job or job security, necessitating the learning of new ways of work [10]. Uncertainty costs as causes of user resistance include uncertainty itself [6] and fear [11]. Loss of value of marketable skills has also been identified as a reason for user resistance, and it is defined as a sunk cost. Similar studies on IS have discussed user resistance [12] and some have focused on the potential reasons for user resistance [11]. In this study, we apply SQB as a base to explain user resistance.

### Behavior model of IT usage

TAM [13] related studies have identified consistent relationships among perceived ease of use (PEOU), perceived usefulness (PU), and behavioral intention (BI), and they typically focus on the decision about whether to use a system. Bhattacherjee and Hikmet show that one of the factors that can influence the BI to use or not use a system is “resistance to use” [2]. Their research provided evidence that “perceived threats” have a major inference on RTC and also that RTC is one of the factors exerting a major effect on the constructs of the TAM. Since the study was performed for a specific profession and the “threats,” the cause of the resistance, cannot fully explain the RTC. Therefore, the effect of resistance on user behavior needs deeper study.

Summarizing from the above related literatures, this study adopts the SQB perspective and combines the IS usage model and Technology Acceptance Model as theoretical foundation to investigates the causes and impacts of users’ RTC on their intention to use the DDS and validates the proposed model with empirical data obtained through survey.

## Methods

### Research scenario, subjects, and sampling

The medical/social service staff used to access applicants’ disability or deciding their welfare level by paper-based simple calculation system. These decision makers are mostly employed by hospitals or local government, such as cities or counties bureau. Those who work in hospitals are responsible for handling the patients’ disability requirement referred by their physicians. Most of them are staff of Physical therapy, Occupational therapy, Nurse, Physician, or Social work departments. They have the chances to face with patients directly and therefore have higher workloads in determining applicants’ disability and welfare levels. Those who work in local governments are officers of health, social affair, or administrative bureau. They are responsible for mostly low income applicants with disability requirement and may need to approach the applicants at their houses or communities to determine their disability and welfare level.

After Taiwan government reformed the disability welfare policies, the disability determination system (DDS) was designed to help the decisions makers the new disability determination process. This system covers the entire determination workflow and is used by various organizations. Disability determination workflow can be divided into four parts: 1) application, 2) execution, 3) audit of the results, and 4) needs assessment based on the results of the disability determination and issuance of the disability welfare certification. The DDS provides disability and social welfare assessment rules for each part of the workflow processes. Using the DDS is mandatory and it serves users across multiple professions, including medical professionals, social workers, and government employees in hospitals, local or central government units.

### Research model and hypotheses development

**Fig. 1:**
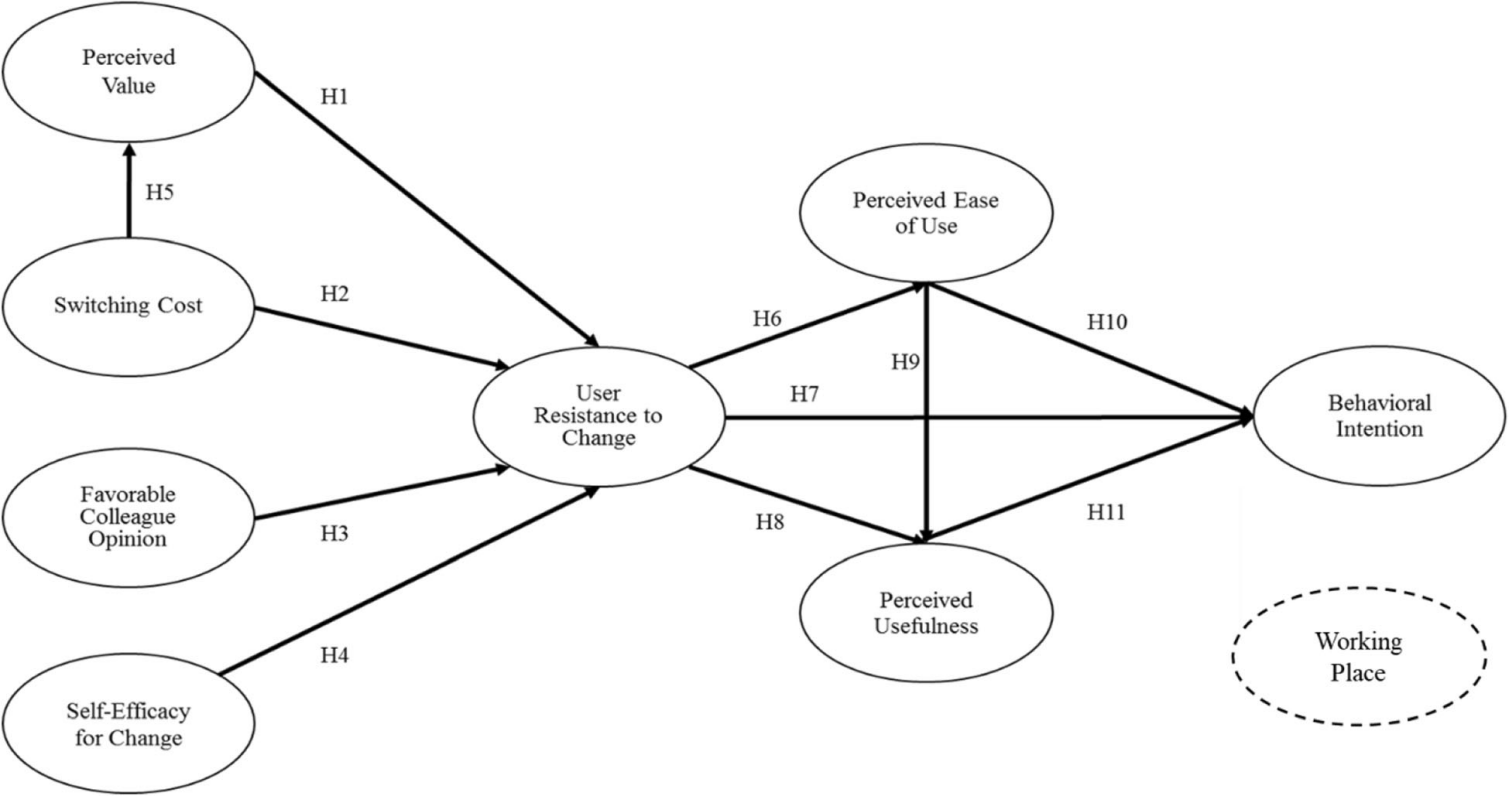
Research model

We developed a research model to fulfill the purposes of this study and integrated resistance as an inhibiter into the TAM. According to Laumer [14], resistance can be a belief, an attitude, or BI in the context of IS research. In this study, we treat resistance as a belief toward the change in the IS. The research model is shown in Fig. 1.

We propose that resistance is the consequence of the SQB, which impacts behavioral beliefs and intentions toward using a new system. This has been implied in prior research that discusses the consequences of engrained habits [6, 8]. In this study, we explicitly hypothesized or formally tested the relationship between resistance and the SQB. In other words, we expect perceived value, favorable colleague opinion, self-efficacy, transition costs, and uncertainty costs to have an impact on PEOU, PU, and intention to use DDS only to the extent to which they bias the user toward the resistance.

Perceived value is defined as the perceived net benefits of change related to the new DDS [6]. In other words, in determining perceived value, rational decision making entails an assessment of whether the benefits that come from the change are worth the costs of the change, thereby bringing SQB into play. If the benefits are thought of as being greater than the costs, users are assumed exhibit lower resistance to the new system. On the other hand, if the perceived value is low, users will have greater RTC. Generally, users will maximize value when they are doing the decision making [15] and are less likely to resist changes when they perceive a higher value [16, 17]. Hence, we propose H1:

H1: User’s perceived value of the DDS has a negative effect on his/her RTC of the system.

In the absence of resistance, it is possible that a user of an old system may readily recognize the advantages of changing to the new system and would form true intentions to do so. Similarly, an individual may perceive high switching costs, but unless these produce resistance, they may readily recognize the relative benefits of the new system and form intentions to switch to it [8]. Thus, we posit the following:

H2. User’s perceived Switching costs of the DDS have a positive effect on his/her RTC of the system.

Colleagues’ opinions have been identified as a salient social influence in the work environment [18]. In this study, colleague opinion was defined as the perception that colleagues favor the changes related to a new IS implementation. Users tend to conform to their coworkers’ opinions because of their need for social friendship [18, 19], and this is termed as normative influence. Thus, we presume that colleagues’ favorable opinions toward changes in the IS may lower user RTC.

H3: Favorable colleague opinion about the DDS has a negative effect on user RTC of the system.

Self-efficacy for change is considered to be an internal factor that can enhance feelings of control. We define self-efficacy for change as people’s belief in their own ability to adapt to a new situation. The problems faced during changes in the IS will appear as challenges to be managed, depending on the individual’s self-efficacy. Users with high self-efficacy will find it easy to adapt to the change. However, users with low self-efficacy will become discouraged and may be more prone to resist the change. Therefore, as self-efficacy decreases, the level of user resistance may increase.

H4: User’s Self-efficacy for change has a negative effect on his/her RTC of the DDS.

The indirect effects of the four subtypes of switching costs, namely, uncertainty costs, transition costs, and sunk costs, are mediated by perceived value. According to the equity implementation model and rational decision making in the context of the SQB, users typically assess net benefits by comparing the costs and benefits of change [20]. Implementing IS brings benefits, such as good performance, and entails costs, such as loss of user control and the time and effort required to learn new workflows. When the costs increase, the net benefits decrease. Therefore, the four subtypes of switching costs reduce the perceived value of new IS implementation and workflow change. Our study includes only three of the four subtypes of switching costs. Therefore, we hypothesize:

H5: User’s perceived Switching costs of the DDS have a negative effect on perceived value of the system.

The indirect effects of inhibitors, such as RTC, will influence enablers, such as perceived usefulness and perceived ease of use, in a negative manner [2]. An inertial user with resistance will perceive lowered perceptions of the new system’s benefits. In other words, resistance will lower the user’s intention to use [8, 17, 21] and affect his/her judgments of PU and PEOU [8]. There are two reasons for this biasing effect. First, norm theory suggests that negative perceptions and acts garner more cognitive attention, are remembered better, and instigate greater information processing than positive ones. Second, inhibitors, when present, tend to anchor one’s overall perception toward attitude objects, subsequently biasing all other perceptions, including those of enablers [22]. In other words, once resistance has arisen, people are not likely to use the new system on account of their perceptions of its usefulness and ease of use. Thus, based on this biasing effect, we propose the following hypothesis:

H6: User’s RTC will negatively impact his/her perceptions of the ease of use of the DDS.

H7: User’s RTC will impact his/her BI toward the DDS.

H8: User’s RTC will negatively impact his/her perceptions of the usefulness of the DDS.

PEOU was found to influence intention to use the IS directly and indirectly via PU. The relationship between PU and PEOU is proved to be positive [13, 23, 24]. Therefore, we propose the following hypothesis:

H9: User’s PEOU of the DDS is positively related to his/her PU of the system.

H10: User’s PEOU of the DDS will impact his/her BI toward the system.

H11: User’s PU of the DDS will impact his/her BI toward the system.

As aforementioned, the users of DSS in different working place may have different work load of determining the applicants’ disability level and diverse tasks to perform. Therefore, working place is used as a control variable in this study.

### Instrument development

The DDS is used by 236 certified hospitals and 363 government departments. Each unit needs 2 to 5 individuals to operate this system depends of the numbers of applications. We firstly obtained the telephone numbers of these organizations from the government website and invited the employees who are in charge of the disability determinations to participate this study. For those who accepted the invitation we then ask for their email address and mail the questionnaire to them.

The survey instrument was a questionnaire developed from a literature review on RTC and TAM. The first draft of the questionnaire consisted of two parts: demographic data and measurement items. There were 6 items in the first part and 38 items in the second part. The scale of items in this study was measured using a 7-point Likert scale, ranging from strongly disagree [19] to strongly agree [22]. The existing validated scales and empirical procedures were adapted to our purpose.

To ensure content validity, two rounds of expert panels were held including two professions in the related areas, one government officer, and two senior workers in the disability determination fields. Then, 5 social workers at a nearby hospital were invited to take pretest, and their feedback was acquired for wording clarity, length of the instrument, and format of the scales. The reliability of this study is reasonable, all the Cronbach’s Alphas are greater than 0.77 (Please see Table 1).

**Table 1:** Reliability and Validity analyses

Variable	Cronbach’s Alpha	Composite Reliability (CR)	Average Variance Extracted (AVE)
BI	0.869	0.937	0.882
FCO	0.927	0.953	0.872
PEOU	0.876	0.910	0.670
PU	0.909	0.943	0.847
PV	0.887	0.912	0.721
RTC	0.937	0.952	0.799
SC	0.859	0.899	0.641
SEC	0.770	0.772	0.647

Convergent validity presents the extent to which the scale correlates positively with other measures of the same construct. Three criteria have been suggested to evaluate convergent validity [25]: (1) Factor loadings of all standardized items should exceed 0.5 and be significant, (2) the composite reliability (CR) should be greater than 0.6, and (3) the average variance extracted (AVE) should exceed 0.5. The loadings of all items in this study are larger than 0.5 and significant below 0.001 except sec_2, therefore the items is deleted. All AVEs exceed 0.64, and all CRs exceed 0.77. Thus, the scale has a good convergent validity. Furthermore, we used the Harman’s single factor test to address the common method variance, since it was easy to use and widely used. All items accounted for less 50% variance; therefore, no common method bias existed in this study.

## Results

### Response rate and representatives

Ethical approval for the study was gained from the Joint Institutional Review Board at Taipei Medical University– IRB serial number: No. 201004001 and No. 201205042. A total of 326 online questionnaires were collected from 22 local governments and 142 hospitals. There were seventy four incomplete responds; therefore, 252 valid samples were used for further analysis. The respondents are majorly female (79.8%), age of 25–34 years (57%), with occupation as social workers, held a Bachelor’s degree (64.9%) and worked at a hospital (67.8%), had less than 1 year of user experience in the disability field (69.4%). The detailed basic information of respondents is shown in Table 2.

**Table 2:** Demographics of Sample

Measure	Categories	Frequency	Percentage(%)
Age	Below 24	11	4.5
	25–34	138	57.0
	35–44	54	22.3
	45–54	35	14.5
	Above 55	4	1.7
Occupation	Physical therapy	8	3.3
	Occupational therapy	12	5.0
	Social worker	132	54.5
	Nurse	4	1.7
	Physician	1	.4
	Administration Staff	72	29.8
	Others	13	5.4
Education	Below high school	10	4.1
	Associate’s Degree	32	13.2
	Bachelor	157	64.9
	Master and above	43	17.8
Working place	Hospital	164	67.8
	Health department	24	9.9
	Social affair department	48	19.8
	Other departments	5	2.5
System Using experience	3 months and below	13	5
	3–6 months	26	10.7
	6–9 months	28	11.6
	9–12 month	101	41.7
	Over 1 year	74	30.6
Disability working experience	3 months and below	23	9.5
	4 month to 1 year	69	28.5
	1–2 years	71	29.3
	2–5 years	43	17.8
	5–10 years	22	9.1
	10 years and above	14	5.7

### Structural model analysis

Eight of the 11 proposed hypotheses were confirmed. They are H2, H3, H5, H7, H8, H9, H10, and H11.

Our finding shows that user’s perceived switching cost has a positive effect and the favorable colleague opinions about the DDS have a negative effect on their RTC of the system and the RTC further influences their perceived usefulness and intension to use the DDS which confirmed the results of prior studies. The relationships between the key elements of TAM hold the same results as those of Davis [13]; Alharbi and Drew [23]; and Beglaryan et al. [24]. However, we found no significant difference of user’s RTC of the system, regardless his/her perceived value of the DDS is high or low (path coefficient = 0.028, t statistics = 0.318 and *p* value = 0.750) which is different form the results of Ali et al. [16] and Hsieh and Lin [17]. Our user reports that self-efficacy for change has no influence on his/her RTC of the DDS (path coefficient = 0.088, t statistics = 0.994 and p value =0.320). The relationship between RTC and DDS perceived ease of use (path coefficient = − 0.143, t statistics = 1.628 and p value = 0.104) confirmed the results of Bhattacherjee and Hikmet [2]. The detail information is shown in Table 3.

**Table 3:** Path Coefficients of Research Framework

Hypotheses		Path Coefficients	t Statistics	*p* Values
H1	PV - > RTC	0.028	0.318	0.750
H2	SC - > RTC	0.362	5.179	0.000***
H3	FCO - > RTC	−0.224	2.590	0.010**
H4	SEC - > RTC	0.088	0.994	0.320
H5	SC - > PV	−0.416	8.726	0.000***
H6	RTC - > PEOU	−0.143	1.628	0.104
H7	RTC - > BI	−0.251	5.044	0.000***
H8	RTC - > PU	− 0.174	2.684	0.007**
H9	PEOU - > PU	0.611	12.169	0.000***
H10	PEOU - > BI	0.236	3.285	0.001***
H11	PU - > BI	0.473	6.124	0.000***

*p < 0.05, **p < 0.01, ***p < 0.001

**Fig. 2:**
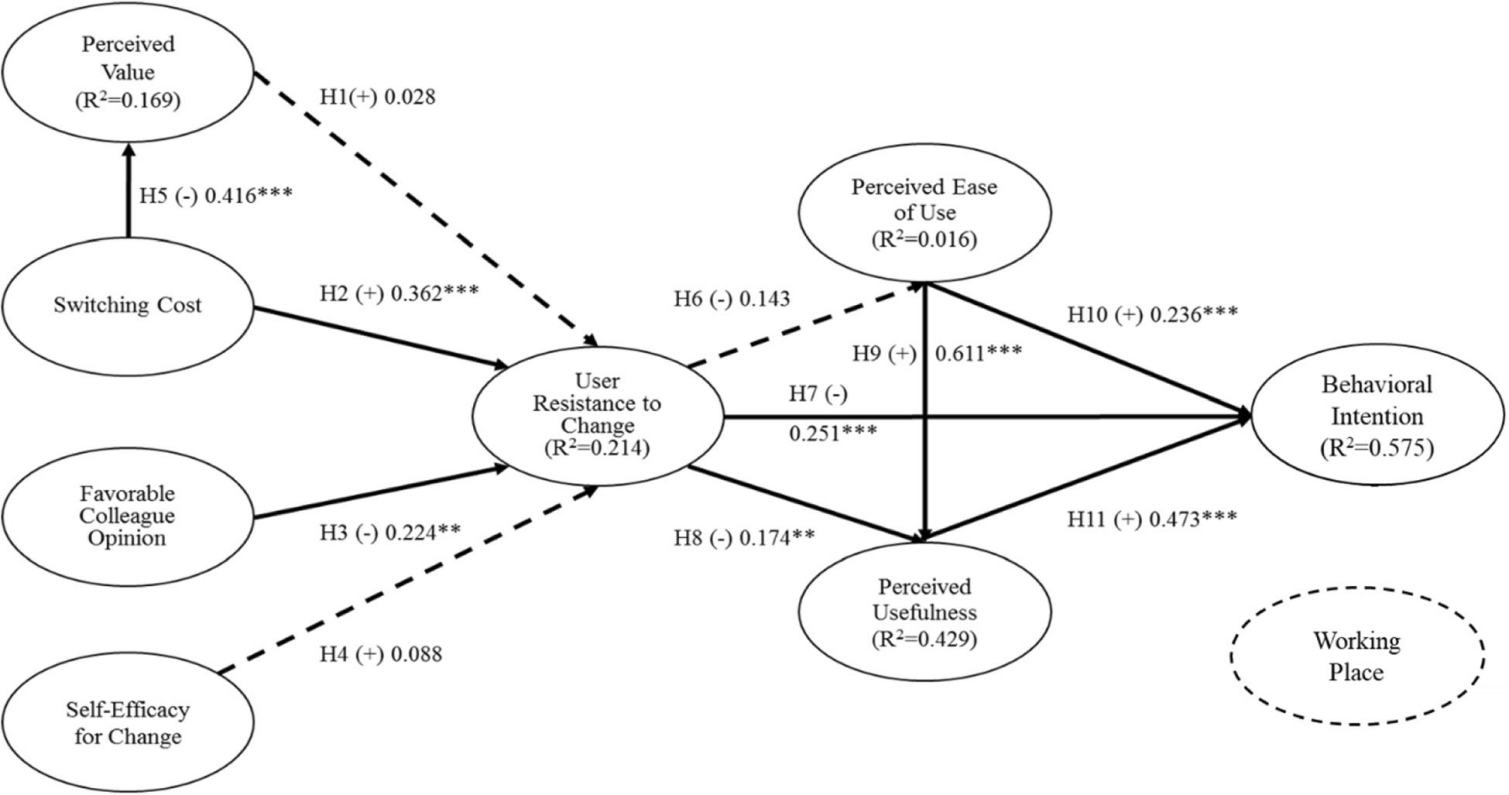
Result of the path analysis

Figure 2 displays the results of the structural model test. The *R*^2^ of PV, PU, PEOU, RTC, perceived value, and BI are 0.169, 0.429, 0.016, 0.214, and 0.575 respectively. Meanwhile, the different working places have indifferent influences on determining the applicants’ disability level and diverse tasks to perform.

## Discussion

Promotion of the new DDS is an important milestone of the Taiwanese government’s informatization polices. However, users’ RTC delays the system implementation process. Unlike most of the prior studies focused on changing healthcare systems within one or multiple case institutions. The DDS is a nationwide disability determination system to evaluate applicants’ level of disability and to determine their deserved warfare level which cannot be customized easily to fit the users in all institutions. The extent of RTC is different from that of a single institution.

The findings of this study have interesting managerial implications. Firstly, the RTC is strongly positive related to “Switch Cost” that users were worried about taking too much time and effort to change to the new DDS and the uncertainty of how the DDS would impact their performance. In other words, manager can lower users’ RTC by lower their switching cost. A well designed training sessions with sufficient contents can guide user step by step and reduce time and effort for learning the new system, reduce the uncertainty and fear, and link users’ prior skill/capability to reduce sunk cost. Secondly, the RTC is strongly negative related to “Favorable Colleague Opinion”. In other words, motivating those who adopted changes of the DDS well to influence their colleagues can reduce RTC substantially among other users. Thirdly, the RTC had no relationship with “Perceived Value” of the DDS. Managers need to decide adequate declaration of the “Perceived Value” of the DSS.

This study revealed that no matter users’ perceived value of the DSS was high or low, their RTC to DDS were the same. Similarly, regardless users’ perceived “Self-efficacy for change” were high or low, their RTC to the DDS were the same. The results of this study can be used by government policy makers to reduce RTC while updating their information system and enhance the effect of the new system. Practical suggestions are two folds. To enhance user intention, the fundamental requirement is to assure that vendors provide a system with correct functions and friendly interface of using the system. To reduce users’ RTC, holding more training sessions with rewards at different time slots can gain earlier adopters. Furthermore, providing an online help desk or hot line to guide users step by step whenever they were stuck in the middle of operating the system would lower down their switching cost and reduce their RTC enormously.

## Conclusion

A theoretical framework can be used to describe, predict, explain, and control phenomena thoroughly. This study combined the SQB, RTC, and TAM as theoretical foundation to explore the impact of RTC on the key components of TAM, PU, PEOU, and BI and reveal more managerial perspectives of RTC for management decision makers while changing their IS. The SQB can reasonably explain the variation or RTC. This study also successfully identified the characteristics of DDS users. The majority of frontline medical social service personnel participants were young and highly professionalized females. Identifying the characteristics of DDS users can help manager understand the frontline medical social service personnel and generate Favorable Colleague Opinion to accommodate the group’s habits.

### Limitations and suggestions to future research

This study developed a model to explain how the SQB affects the RTC and how RTC influences BI. A number of future research avenues could be followed based on this study. Although our model explained 58% of the variance of BI, it is necessary to discover other factors that may contribute to the BI of the DDS. Second, the model we proposed was cross-sectional. The user’s perceptions were measured at a single point of time. However, perceptions may change over time as people obtain more experiences. Therefore, a longitudinal study exploring the changing status of SQB dimensions over time would be useful. Third, applying the model proposed in this study to different subjects and different working processes in disability determination may shed light on new aspects of DDS usage.

## Data Availability

The datasets used and/or analyzed during this study are available from the corresponding author on reasonable request.

## Abbreviations

RTC: Resistance to Change
IS: Information Systems
IT: Information Technology
DDS: Disability Determination System
SQB: Status Quo Bias
TAM: Technology Acceptance Model
PEOU: Perceived Ease of Use
PU: Perceived Usefulness
BI: Behavioral Intention
SC: Switching Cost
SEC: Self-Efficacy for Change
FCO: Favorable Colleague Opinion
